# Cognitive composites for genetic frontotemporal dementia: GENFI-Cog

**DOI:** 10.1186/s13195-022-00958-0

**Published:** 2022-01-19

**Authors:** Jackie M. Poos, Katrina M. Moore, Jennifer Nicholas, Lucy L. Russell, Georgia Peakman, Rhian S. Convery, Lize C. Jiskoot, Emma van der Ende, Esther van den Berg, Janne M. Papma, Harro Seelaar, Yolande A. L. Pijnenburg, Fermin Moreno, Raquel Sanchez-Valle, Barbara Borroni, Robert Laforce, Mario Masellis, Carmela Tartaglia, Caroline Graff, Daniela Galimberti, James B. Rowe, Elizabeth Finger, Matthis Synofzik, Rik Vandenberghe, Alexandre de Mendonça, Pietro Tiraboschi, Isabel Santana, Simon Ducharme, Chris Butler, Alexander Gerhard, Johannes Levin, Adrian Danek, Markus Otto, Isabel Le Ber, Florence Pasquier, John C. van Swieten, Jonathan D. Rohrer, on behalf of the Genetic FTD Initiative (GENFI), Arabella Bouzigues, Martin N. Rossor, Nick C. Fox, Jason D. Warren, Martina Bocchetta, Imogen J. Swift, Rachelle Shafei, Carolin Heller, Emily Todd, David Cash, Ione Woollacott, Henrik Zetterberg, Annabel Nelson, Rita Guerreiro, Jose Bras, David L. Thomas, Simon Mead, Lieke Meeter, Jessica Panman, Rick van Minkelen, Myriam Barandiaran, Begoña Indakoetxea, Alazne Gabilondo, Mikel Tainta, Ana Gorostidi, Miren Zulaica, Alina Díez, Jorge Villanua, Sergi Borrego-Ecija, Jaume Olives, Albert Lladó, Mircea Balasa, Anna Antonell, Nuria Bargallo, Enrico Premi, Stefano Gazzina, Roberto Gasparotti, Silvana Archetti, Sandra Black, Sara Mitchell, Ekaterina Rogaeva, Morris Freedman, Ron Keren, David Tang-Wai, Hakan Thonberg, Linn Öijerstedt, Christin Andersson, Vesna Jelic, Andrea Arighi, Chiara Fenoglio, Elio Scarpini, Giorgio Fumagalli, Thomas Cope, Carolyn Timberlake, Timothy Rittman, Christen Shoesmith, Robart Bartha, Rosa Rademakers, Carlo Wilke, Hans-Otto Karnarth, Benjamin Bender, Rose Bruffaerts, Philip Vandamme, Mathieu Vandenbulcke, Catarina B. Ferreira, Gabriel Miltenberger, Carolina Maruta, Ana Verdelho, Sónia Afonso, Ricardo Taipa, Paola Caroppo, Giuseppe Di Fede, Giorgio Giaccone, Sara Prioni, Veronica Redaelli, Giacomina Rossi, Diana Duro, Maria Rosario Almeida, Miguel Castelo-Branco, Maria João Leitão, Miguel Tabuas-Pereira, Beatriz Santiago, Serge Gauthier, Pedro Rosa-Neto, Michele Veldsman, Paul Thompson, Tobias Langheinrich, Catharina Prix, Tobias Hoegen, Elisabeth Wlasich, Sandra Loosli, Sonja Schonecker, Sarah Anderl-Straub, Jolina Lombardi, Nuria Bargalló, Alberto Benussi, Valentina Cantoni, Maxime Bertoux, Anne Bertrand, Alexis Brice, Agnès Camuzat, Olivier Colliot, Sabrina Sayah, Aurélie Funkiewiez, Daisy Rinaldi, Gemma Lombardi, Benedetta Nacmias, Dario Saracino, Valentina Bessi, Camilla Ferrari, Marta Cañada, Vincent Deramecourt, Gregory Kuchcinski, Thibaud Lebouvier, Sebastien Ourselin, Cristina Polito, Adeline Rollin

**Affiliations:** 1 Department of Neurology, Erasmus MC University Medical Center, Rotterdam, The Netherlands; 2 Dementia Research Centre, Department of Neurodegenerative Disease, National Hospital for Neurology and Neurosurgery, UCL Institute of Neurology, 8-11 Queen Square, Box 16, London, WC1N 3BG UK; 3 Department of Medical Statistics, London School of Hygiene and Tropical Medicine, London, UK; 4 Department of Neurology, Alzheimer Center, Amsterdam University Medical Center, Amsterdam Neuroscience, Amsterdam, The Netherlands; 5 Cognitive Disorders Unit, Department of Neurology, Donostia University Hospital, San Sebastian, Gipuzkoa Spain; 6 Alzheimer’s disease and Other Cognitive Disorders Unit, Neurology Service, Hospital Clínic, Institut d’Investigacións Biomèdiques August Pi I Sunyer, University of Barcelona, Barcelona, Spain; 7 Centre for Neurodegenerative Disorders, Neurology Unit, Department of Clinical and Experimental Sciences, University of Brescia, Brescia, Italy; 8 Clinique Interdisciplinaire de Mémoire, Département des Sciences Neurologiques, Université Laval, Québec, Canada; 9 Sunnybrook Health Sciences Centre, Sunnybrook Research Institute, University of Toronto, Toronto, Canada; 10 Tanz Centre for Research in Neurodegenerative Diseases, University of Toronto, Toronto, Canada; 11 Department of Geriatric Medicine, Karolinska University Hospital-Huddinge, Stockholm, Sweden; 12 University of Milan, Centro Dino Ferrari, Milan, Italy; 13 Neurodegenerative Diseases Unit, Fondazione IRCCS Ca’ Granda, Ospedale Policlinico, Milan, Italy; 14 Department of Clinical Neurosciences, University of Cambridge, Cambridge, UK; 15 Department of Clinical Neurological Sciences, University of Western Ontario, London, Ontario Canada; 16 Department of Neurodegenerative Diseases, Hertie-Institute for Clinical Brain Research and Center of Neurology, University of Tübingen, Tübingen, Germany; 17 German Center for Neurodegenerative Diseases (DZNE), Tübingen, Germany; 18 Laboratory for Cognitive Neurology, Department of Neurosciences, KU Leuven, Leuven, Belgium; 19 Faculty of Medicine, University of Lisbon, Lisbon, Portugal; 20 Fondazione Istituto di Ricovero e Cura a Carattere Scientifico Istituto Neurologica Carlo Besta, Milan, Italy; 21 Faculty of Medicine, University of Coimbra, Coimbra, Portugal; 22 Department of Psychiatry, McGill University Health Centre, McGill University, Montreal, Québec Canada; 23 Department of Clinical Neurology, University of Oxford, Oxford, UK; 24 Faculty of Medical and Human Sciences, Institute of Brain, Behaviour and Mental Health, University of Manchester, Manchester, UK; 25 Department of Neurology, Ludwig-Maximilians-University, Munich, Germany; 26 German Center for Neurodegenerative Diseases (DZNE), Munich, Germany; 27 Munich Cluster for Systems Neurology (SyNergy), Munich, Germany; 28 Department of Neurology, University of Ulm, Ulm, Germany; 29 Paris Brain Institute – Institut du Cerveau – ICM, Inserm U1127, CNRS UMR 7225, AP-HP - Hôpital Pitié-Salpêtrière, Sorbonne Université, Paris, France; 30 Centre de référence des démences rares ou précoces, IM2A, Département de Neurologie, AP-HP - Hôpital Pitié-Salpêtrière, Paris, France; 31 Département de Neurologie, AP-HP - Hôpital Pitié-Salpêtrière, Paris, France; 32 University of Lille, Lille, France; 33 Inserm 1172, Lille, France; 34 CHU, CNR-MAJ, Labex Distalz, LiCEND, Lille, France

**Keywords:** Frontotemporal dementia, Cognition, Neuropsychology, Composite score, Language, Attention, Executive function, Memory, Social cognition

## Abstract

**Background:**

Clinical endpoints for upcoming therapeutic trials in frontotemporal dementia (FTD) are increasingly urgent. Cognitive composite scores are often used as endpoints but are lacking in genetic FTD. We aimed to create cognitive composite scores for genetic frontotemporal dementia (FTD) as well as recommendations for recruitment and duration in clinical trial design.

**Methods:**

A standardized neuropsychological test battery covering six cognitive domains was completed by 69 *C9orf72*, 41 *GRN*, and 28 *MAPT* mutation carriers with CDR® plus NACC-FTLD ≥ 0.5 and 275 controls. Logistic regression was used to identify the combination of tests that distinguished best between each mutation carrier group and controls. The composite scores were calculated from the weighted averages of test scores in the models based on the regression coefficients. Sample size estimates were calculated for individual cognitive tests and composites in a theoretical trial aimed at preventing progression from a prodromal stage (CDR® plus NACC-FTLD 0.5) to a fully symptomatic stage (CDR® plus NACC-FTLD ≥ 1). Time-to-event analysis was performed to determine how quickly mutation carriers progressed from CDR® plus NACC-FTLD = 0.5 to ≥ 1 (and therefore how long a trial would need to be).

**Results:**

The results from the logistic regression analyses resulted in different composite scores for each mutation carrier group (i.e. *C9orf72*, *GRN*, and *MAPT*). The estimated sample size to detect a treatment effect was lower for composite scores than for most individual tests. A Kaplan-Meier curve showed that after 3 years, ~ 50% of individuals had converted from CDR® plus NACC-FTLD 0.5 to ≥ 1, which means that the estimated effect size needs to be halved in sample size calculations as only half of the mutation carriers would be expected to progress from CDR® plus NACC FTLD 0.5 to ≥ 1 without treatment over that time period.

**Discussion:**

We created gene-specific cognitive composite scores for *C9orf72*, *GRN*, and *MAPT* mutation carriers, which resulted in substantially lower estimated sample sizes to detect a treatment effect than the individual cognitive tests. The GENFI-Cog composites have potential as cognitive endpoints for upcoming clinical trials. The results from this study provide recommendations for estimating sample size and trial duration.

**Supplementary Information:**

The online version contains supplementary material available at 10.1186/s13195-022-00958-0.

## Background

Frontotemporal dementia (FTD) encompasses a heterogeneous group of early-onset neurodegenerative disorders caused by prominent frontal and/or temporal lobe degeneration with a wide range of overlapping clinical features [1]. The two main phenotypes are behavioural variant FTD (bvFTD), with prominent behavioural changes and executive dysfunction [2], and primary progressive aphasia (PPA), with impairment in language comprehension and/or production [3]. FTD is a highly heritable disease, with 20–30% of cases having an autosomal dominant pattern of inheritance [4]. The most common causes of genetic FTD are mutations in the microtubule-associated protein tau (*MAPT*), progranulin (*GRN*), and chromosome 9 open reading frame 72 (*C9orf72*) genes [4].

Clinical trials testing disease-modifying treatments for FTD are now underway, and clinical endpoints to monitor treatment response are therefore urgently needed. It is believed that interventions may have the most profound effect if initiated in the earliest stages of the disease; however, a major challenge facing these clinical trials is the lack of outcome measures that are sensitive enough to track the effects of treatment in the early stages of the disease [5–7].

Traditional outcomes such as progression to clinical diagnosis or cognitive measures developed for other forms of dementia such as Alzheimer’s disease (AD) might not be well-suited to serve as endpoints for early-stage FTD treatment trials because of the large sample size and long trial duration that would be required to measure possible treatment effects or due to the psychometric properties of the tests themselves [8–10]. Sensitive outcome measures in patients with clinically diagnosed AD, such as the Alzheimer’s Disease Assessment Scale Cognitive Subscale (ADAS-Cog), might not be sensitive to decline in patients with FTD [10, 11]. Multiple genetic FTD cohort studies have investigated a wide range of cognitive instruments and found gene-specific cognitive impairment and/or decline in language, executive function, social cognition, attention/processing speed, and memory, in symptomatic and presymptomatic stages [12–27]. However, due to the subtlety of cognitive decline in the early stages of the disease, using individual tests as outcome measures might not be sensitive enough to detect a treatment effect. Furthermore, an individual cognitive test is limited to measuring only one specific symptom, and due to the heterogeneity of clinical features between FTD patients, tests from multiple cognitive domains would need to be included. A selection of the most sensitive tests for each genetic group would enable shortening of the neuropsychological test battery thereby significantly minimizing time and other resource costs compared to using a broad range of individual cognitive tests [28].

Composite scores are often used in clinical trials to reduce the number of variables used as outcome measures [8]. A composite score is any measure which combines the results of multiple cognitive and clinical assessments into a single summary score [29]. As a result, it provides a measure of multiple domains but can serve as a single primary endpoint in clinical trials [8]. Such composites have been developed for several neurodegenerative disorders, such as AD (e.g. the ADAS-Cog [11]), Parkinson’s disease (PD) (e.g. the Unified Parkinson’s Disease Rating Scale (UPDRS) [30]), and Huntington’s disease (HD) (e.g. the Unified Huntington’s Disease Rating Scale (UHDRS) [29]) but are, as of yet, lacking in FTD.

Therefore, the aim of this study was to create gene-specific cognitive composite scores for *MAPT*, *GRN*, and *C9orf72* mutation carriers in the early symptomatic stage by empirically determining the combination of neuropsychological tests most sensitive to differentiate mutation carriers from non-carriers. Data was collected within the Genetic FTD Initiative (GENFI), an international genetic FTD cohort study aimed at developing novel markers of disease onset and progression [14]. To evaluate their performance, we compared the sample size requirements between each of the proposed composites and individual cognitive tests for a theoretical trial aimed at preventing progression from a prodromal stage (CDR® plus NACC-FTLD [31] = 0.5) to a fully symptomatic stage (CDR® plus NACC-FTLD ≥ 1). Lastly, we performed time-to-event analyses to determine how many people progressed from a CDR® plus NACC FTLD 0.5 to ≥ 1, to provide recommendations on the duration of such clinical trials.

## Methods

### Participants

Data was included from the fifth GENFI data freeze in which participants from confirmed genetic FTD families were recruited between 30 January 2012 and 31 May 2019 in 24 centres across Europe and Canada. A total of 69 *C9orf72*, 41 *GRN*, and 28 *MAPT* mutation carriers with a CDR® plus NACC FTLD ≥ 0.5 and 275 mutation-negative controls (i.e. family members who tested negative for the mutation) were included in this study. Of the mutation carrier group, 41 *C9orf72*, 17 *GRN*, and 16 *MAPT* mutation carriers fulfilled the diagnostic criteria for bvFTD [2] (*C9orf72* = 36, *GRN* = 11, *MAPT* = 16), PPA [3] (*GRN* = 6), or FTD with amyotrophic lateral sclerosis (FTD-ALS) [32] (*C9orf72* = 5). Participant characteristics are summarized in Table 1, and the number of participants included in each of the statistical analysis steps can be found in Fig. S1.

**Table 1:** Participant characteristics and neuropsychological test results

	***C9orf72***	***GRN***	***MAPT***	Controls
**Number of participants**	69	41	28	275
**Sex, f:m**	30:39	20:21	14:14	160:115
**Age**	55 (12.0)	53.0 (11.4)	51.1 (12.6)	45.8 (12.7)
**Education**	13.7 (3.1)	14.0 (3.5)	14.3 (3.4)	14.6 (3.4)
**MMSE**	27.1 (3.2)	26.6 (7.0)	27.5 (3.0)	29.3 (2.1)
**CDR® plus NACC FTLD sob**	5.9 (5.5)	3.4 (4.8)	4.8 (5.0)	0.2 (0.6)
**Language**				
**Camel and Cactus Test**	− 1.81 (2.81)	− 0.57 (1.36)	− 2.10 (3.08)	–
**Boston Naming Test**	− 1.77 (3.32)	− 0.68 (1.62)	− 2.63 (3.16)	–
**Category fluency**	− 1.20 (1.05)	− 0.54 (1.04)	− 0.84 (1.14)	–
**Attention and mental processing speed**				
**Digit span forward**	− 0.39 (1.19)	− 0.08 (1.26)	0.13 (1.23)	–
**Trail Making Test – part A**	− 1.37 (2.17)	− 0.69 (1.63)	− 0.72 (1.54)	–
**Digit symbol**	− 1.18 (1.30)	− 0.62 (1.23)	− 0.67 (1.31)	–
**D-KEFS CWIT – colour naming**	− 2.85 (3.58)	− 0.52 (1.85)	− 1.30 (2.17)	–
**D-KEFS CWIT – word naming**	− 1.86 (3.11)	− 0.02 (1.46)	− 0.54 (1.47)	–
**Executive function**				
**Digit span backward**	− 0.53 (1.23)	− 0.49 (1.23)	− 0.19 (0.98)	–
**Trail Making Test – part B**	− 2.44 (2.95)	− 1.81 (3.06)	− 1.37 (2.58)	–
**D-KEFS CWIT – ink naming**	− 3.46 (3.91)	− 1.13 (2.21)	− 1.16 (2.54)	–
**Phonemic fluency**	− 1.18 (1.18)	− 0.08 (1.33)	− 0.64 (1.28)	–
**Visuoconstruction**				
**Benson figure copy**	− 0.90 (1.90)	− 0.06 (1.16)	− 0.46 (1.39)	–
**Memory**				
**Benson figure recall**	− 0.72 (1.57)	− 0.75 (1.46)	− 1.27 (1.91)	–
**FCSRT free recall**	− 1.68 (1.36)	− 0.72 (1.49)	− 1.71 (1.80)	–
**FCSRT total recall**	− 2.20 (3.56)	− 1.42 (3.05)	− 2.86 (3.62)	–
**FCSRT delayed free recall**	− 1.59 (1.59)	− 0.97 (1.58)	− 1.72 (2.04)	–
**FCSRT delayed total recall**	− 2.10 (3.81)	− 1.13 (3.09)	− 2.82 (4.02)	–
**Social cognition**				
**Facial Emotion Recognition Test**	− 1.67 (1.87)	− 1.00 (1.47)	− 1.04 (1.59)	–

Values are mean *Z*-scores (raw score − mean score controls/standard deviation of controls) corrected for age, years of education, and sex, with standard deviation in parentheses unless otherwise specified. For the FCSRT and letter fluency, an additional correction was made for language as stimuli differed between languages

*Abbreviations*: *C9orf72* chromosome 9 open reading frame 72, *GRN* progranulin, *MAPT* microtubule-associated protein tau, *MMSE* Mini-Mental State Examination, *CDR® plus NACC FTLD sob* Clinical Dementia Rating scale plus National Alzheimer’s Coordinating Center Frontotemporal Lobar Degeneration sum of boxes, *D-KEFS CWIT* Delis-Kaplan Executive Function System Colour-Word Interference Test, *FCSRT* Free and Cued Selective Reminding Test

### Procedure

All participants completed a comprehensive neuropsychological test battery covering six cognitive domains: language (modified Camel and Cactus Test [33]; Boston Naming Test (BNT, short 30 item version) [34]; category fluency (animals) [35]), attention/processing speed and executive function (WMS-R Digit span [34]; Trail Making Test (TMT) [36]; WAIS-R Digit Symbol test [34]; D-KEFS Colour-Word Interference Test (CWIT) [37]; phonemic fluency [35]), verbal and visuospatial memory (Free and Cued Selective Reminding Test (FCSRT) [20]; Benson figure recall), social cognition (Facial Emotion Recognition test [38]), and visuoconstruction (Benson figure copy). The Mini-Mental State Examination (MMSE) [39] was administered to measure global cognitive functioning, and clinical status was determined by means of a structured clinical interview, including the CDR® plus NACC FTLD [31].

### Statistical methods

Statistical analyses were performed using Stata version 14 and R version 3.6.2. We compared the continuous demographic data between the mutation carrier groups with Kruskal-Wallis and post hoc Mann-Whitney tests. A chi-square test was used to compare sex between the groups.

All neuropsychological data were converted to *Z*-scores corrected for age, education, and sex compared to the control group collected within GENFI (i.e. mutation-negative participants). The FCSRT and letter fluency scores were also corrected for language as the test stimuli differed by language across the different GENFI sites. The control data available in each language can be found in Additional file 1: Table S1. *Z*-scores for tests with reaction times (i.e. TMT and D-KEFS CWIT) were inversed so that lower *Z*-scores indicated worse performance on all tests. A detailed description of how the corrected *Z*-scores were calculated can be found in Additional file 1.

#### Creating the composite scores

Least absolute shrinkage and selection operator (LASSO) [40] logistic regression models with 10-fold cross-validation were used to identify the combination of neuropsychological tests that discriminated best between each mutation carrier group and controls. Participants with missing data were excluded from this analysis. A separate model was fitted for each genetic group with carrier status as the outcome and the neuropsychological tests as the predictors. A detailed description of the statistical methods can be found in Additional file 1. The glmnet package in R was used to fit the LASSO models and carry out the cross-validation.

From the resulting model, two different cognitive composite scores were calculated: (1) an average of the scores for all cognitive tests that were selected in the model and (2) a weighted average of the scores for all cognitive tests that were selected in the model, using the regression coefficients to determine the weights.

#### Sample size calculation

For each outcome, the sample size was calculated for a hypothetical two-arm study with 1:1 randomization to placebo versus active drug with 80% power to detect a treatment effect at a 5% significance level [41]. The focus of future studies is likely to be on treating people with very early symptomatic disease, and so, we focused on calculating the sample sizes for a trial of prodromal mutation carriers (i.e. CDR® plus NACC FTLD = 0.5) where the therapeutic drug had an effect on the progression to being fully symptomatic (i.e. CDR® plus NACC FTLD = 1). We therefore calculated sample sizes for a 10%, 20%, and 40% effect size where a 100% treatment effect would be the difference in the mean between the CDR® plus NACC FTLD 0.5 and 1 groups. Choosing the effect size in this way assumes that the hypothetical treatment will prevent a given proportion of the decline in cognitive scores seen between these two groups. For example, a 20% treatment effect assumes that the untreated group will experience the change seen between CDR® plus NACC FTLD 0.5 and 1 groups, but the treated group will only experience 80% of this change (i.e. 20% less). See Additional file 1 for more details on the sample size calculations and the parameters used (Additional file 1: Table S2) [41].

#### Time-to-event analysis

**Fig. 1:**
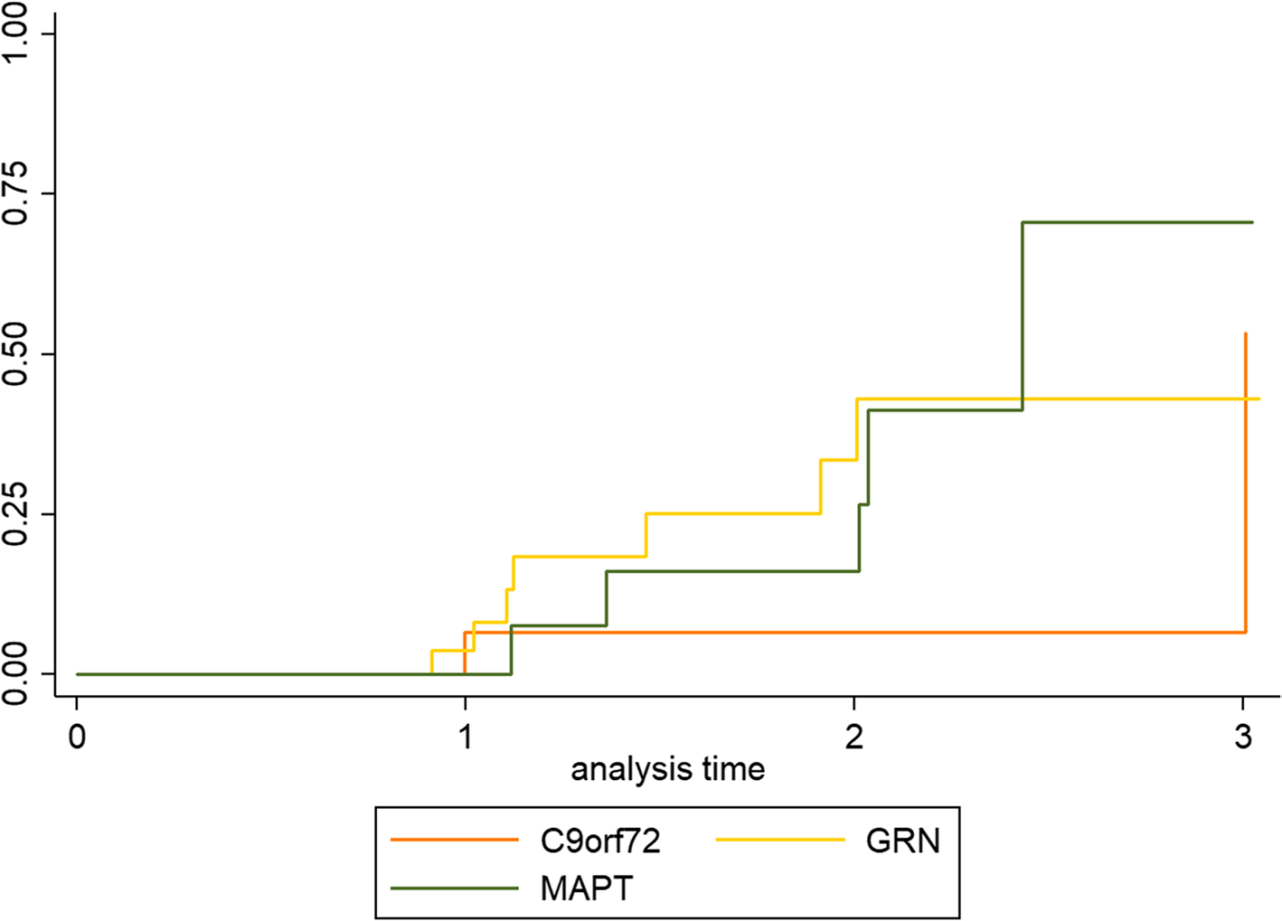
Kaplan-Meier estimates of mutation carriers that converted from CDR® plus NACC FTLD 0.5 to ≥ 1. The number of mutation carriers included, and the number that progressed or were lost to follow-up are reported in Additional file 1: Table S4. C9orf72, chromosome 9 open reading frame 72; GRN, progranulin; MAPT, microtubule-associated protein tau; CDR plus NACC FTLD, Clinical Dementia Rating scale plus National Alzheimer’s Coordinating Center Frontotemporal Lobar Degeneration

To provide recommendations on the timeline for the hypothesized trial, we present Kaplan-Meier curves showing the cumulative proportion of participants who progressed from a CDR® plus NACC FTLD 0.5 to ≥ 1 within the GENFI cohort over time. In this analysis, the censoring date was the date of conversion or the date of the last follow-up. As this is an ongoing prospective cohort study, not all mutation carriers completed all study visits which resulted in missing data. There were 62 mutation carriers (19 *C9orf72*, 27 *GRN*, and 16 *MAPT*) that had a CDR® plus NACC FTLD of 0.5 and one or multiple follow-up visits and were included in the time-to-event analysis (Additional file 1: Table S4 and Fig. 1). A log rank test was performed to compare the rate of progression between the genetic groups.

## Results

### Demographics

Participant characteristics for all mutation carriers are summarized in Table 1. Overall, the number of males to females differed between the groups (*p* = 0.020). *C9orf72*, *GRN*, and *MAPT* mutation carriers were older and had lower MMSE and higher CDR® plus NACC FTLD sum of boxes scores than controls (all *p* < 0.010). In addition, *C9orf72* mutation carriers had higher CDR® plus NACC FTLD sum of boxes scores than *GRN* mutation carriers (*p* = 0.007). There were no differences between the groups in years of education (*p* = 0.290). The characteristics of participants when individually stratified by CDR® plus NACC FTLD global score (i.e. in 0.5, 1, 2, and 3 groups) can be found in Additional file 1: Table S3.

### Logistic regression analyses

**Fig. 2:**
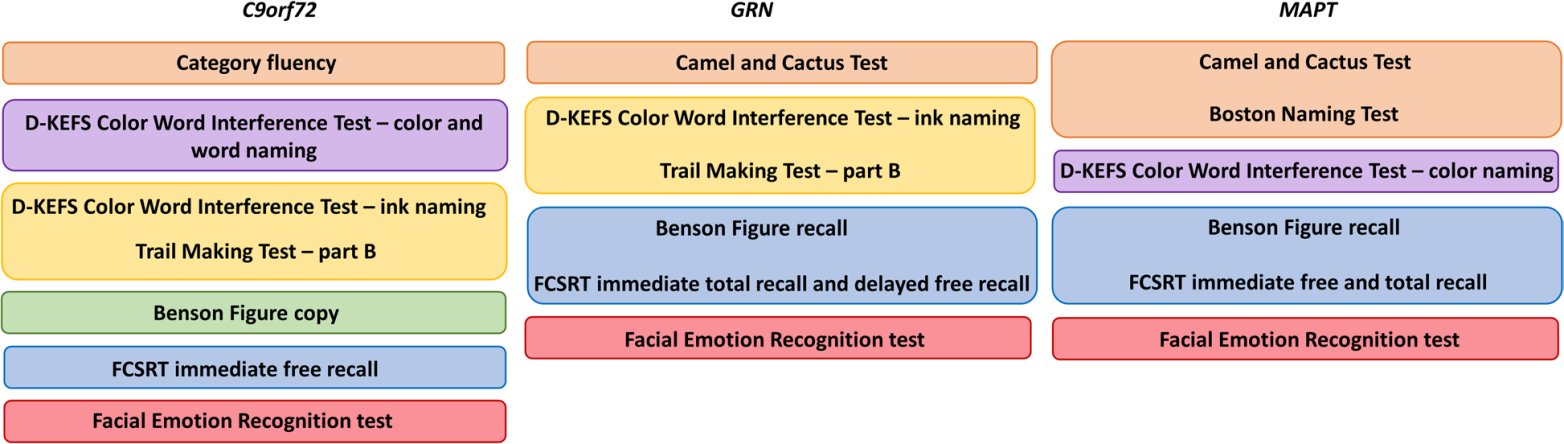
Overview of the neuropsychological tests included in the GENFI-Cog scores per cognitive domain. C9orf72, chromosome 9 open reading frame 72; GRN, progranulin; MAPT, microtubule-associated protein tau

The results from the logistic regression model can be seen in Table 2. A combination of category fluency, D-KEFS CWIT – colour, word and ink naming, TMT – part B, the Benson figure copy, FCSRT free recall, and the Facial Emotion Recognition Test was most sensitive to discriminate *C9orf72* repeat expansion carriers from controls. For *GRN* mutation carriers, a combination of the Camel and Cactus Test, TMT – part B, D-KEFS CWIT – ink naming, Benson figure recall, FCSRT total and delayed free recall, and the Facial Emotion Recognition Test was most sensitive. In *MAPT* mutation carriers, a combination of the Camel and Cactus Test, BNT, D-KEFS CWIT – colour naming, Benson figure recall, FCSRT free, total and delayed free recall, and the Facial Emotion Recognition Test was most sensitive to differentiate from controls. For each mutation carrier group, the average and weighted composite scores were calculated, including the tests with a negative coefficient in Table 2. A summary of the included tests that were included in each GENFI-Cog per gene group can be seen in Fig. 2.

**Table 2:** Regression coefficients and corresponding weights

	***C9orf72***		***GRN***		***MAPT***	
	Coef.	Weight	Coef.	Weight	Coef.	Weight
**Language**						
**Camel and Cactus Test**			− 0.004	0.003	− 0.04	0.04
**Boston Naming Test**					− 0.39	0.40
**Category fluency**	− 0.13	0.09				
**Attention and mental processing speed**						
**Digit span forward**						
**Trail Making Test – part A**						
**Digit symbol**						
**D-KEFS CWIT – colour naming**	− 0.06	0.04			− 0.09	0.09
**D-KEFS CWIT – word naming**	− 0.04	0.03	0.09^a^			
**Executive function**						
**Digit span backward**						
**Trail Making Test – part B**	− 0.07	0.05	− 0.28	0.23		
**D-KEFS CWIT – ink naming**	− 0.29	0.20	− 0.24	0.20		
**Phonemic fluency**			0.24^a^			
**Visuoconstruction**						
**Benson figure copy**	− 0.09	0.06				
**Memory**						
**Benson figure recall**			− 0.06	0.05	− 0.01	0.01
**FCSRT free recall**	− 0.50	0.35			− 0.06	0.06
**FCSRT total recall**			− 0.05	0.04	− 0.30	0.31
**FCSRT delayed free recall**			− 0.16	0.13	− 0.01	0.01
**FCSRT delayed total recall**						
**Social cognition**						
**Facial Emotion Recognition Test**	− 0.26	0.18	− 0.42	0.35	− 0.08	0.08

Data are presented as coefficients and weights. Coefficient gives the change in log odds of being a mutation carrier for each *Z*-score increase in the score on the cognitive test. Weight gives the weighting used when calculating the weighted cognitive composite score

*Abbreviations*: *C9orf72* chromosome 9 open reading frame 72, *GRN* progranulin, MAPT microtubule-associated protein tau, *D-KEFS CWIT* Delis-Kaplan Executive Function System Colour-Word Interference Test, *FCSRT* Free and Cued Selective Reminding Test

^a^Positive coefficients indicate better performance in mutation carriers compared to controls and were not included in the composite score

### Sample size calculation

Sample size estimates can be observed in Table 3. In *C9orf72* repeat expansion carriers, both the average and weighted composite scores resulted in lower sample sizes than most individual cognitive tests. The only test that resulted in a lower sample size than the composite score was the D-KEFS CWIT – ink naming, with the digit symbol test also resulting in a lower sample size than the average but not the weighted composite score. In *GRN* mutation carriers, again both composite scores resulted in lower sample sizes than for most individual cognitive tests except the TMT – part B. The TMT – part A also resulted in a lower sample size than the weighted composite, but not the average composite. In addition, the D-KEFS CWIT – ink naming resulted in a sample size of less than 100, albeit not lower than the composites. In *MAPT* mutation carriers, both composites resulted in estimated sample sizes smaller than 130 with an effect size of 0.1, but the TMT – part A, digit symbol test, and D-KEFS CWIT – colour and ink naming resulted in even lower sample sizes (*n* < 100). In *C9orf72* and *MAPT* mutation carriers, the weighted composite score resulted in a lower estimated sample size than the average composite, whereas in *GRN* mutation carriers the average composite resulted in a lower sample size. For *GRN* (all *n* < 60) and *MAPT* (all *n* < 125) mutation carriers, lower sample sizes would be necessary to detect a treatment effect than for *C9orf72* repeat expansion carriers (all *n* ≤ 306).

**Table 3:** Sample size per arm for a hypothetical clinical trial using different cognitive outcome measures

Outcome measures	***C9orf72***			***GRN***			***MAPT***		
	ES 10%	ES 20%	ES 40%	ES 10%	ES 20%	ES 40%	ES 10%	ES 20%	ES 40%
**Cognitive composite scores**									
**Average composite**	306	76	19	27	7	2	124	31	8
**Weighted composite**	214	53	13	53	13	3	90	23	6
**Language**									
**Camel and Cactus Test**	4946	1237	309	292	73	18	357	89	22
**Boston Naming Test**	1109	277	69	213	53	13	223	56	14
**Category fluency**	1584	396	99	781	195	49	400	100	25
**Attention and mental processing speed**									
**Digit span forward**	13,0210	32,553	8138	2677	669	167	17,773	4443	1111
**Trail Making Test – part A**	2272	568	142	45	11	3	69	17	4
**Digit symbol**	254	64	16	925	231	58	80	20	5
**D-KEFS CWIT – colour naming**	866	216	54	502	126	31	66	17	4
**D-KEFS CWIT – word naming**	19,224	4806	1202	3310	828	207	150	37	9
**Executive functioning**									
**Digit span backward**	1724	431	108	840	210	52	26,218	6555	1639
**Trail Making Test – part B**	1275	319	80	25	6	2	81	20	5
**D-KEFS CWIT – ink naming**	61	15	4	70	17	4	26	7	2
**Phonemic fluency**	558	139	35	2229	557	139	161	40	10
**Visuoconstruction**									
**Benson figure copy**	5911	1478	369	2119	530	132	6,282,036	1,570,509	392,627
**Memory**									
**Benson figure recall**	1044	261	65	657	164	41	7611	1903	476
**FCSRT free recall**	1302	326	81	294	74	18	521	130	33
**FCSRT total recall**	1020	255	64	477	119	30	524	131	33
**FCSRT delayed free recall**	606	152	38	767	192	48	261	65	16
**FCSRT delayed total recall**	358	89	22	193	48	12	681	170	43
**Social cognition**									
**Facial Emotion Recognition Test**	7570	1892	473	7805	1951	488	147	37	9

The sample size per arm was estimated as *n* = (1 − *ρ*^2)(2*σ*^2)/*δ*^2 *f*(*α*,*β*), where *ρ* is the correlation between baseline and follow-up measures of the outcome, *σ* is the standard deviation of the outcome in the CDR® plus NACC-FTLD 0.5 group, *δ* is the treatment effect (effect size multiplied by the difference in mean between CDR® plus NACC-FTLD 0.5 and 1 group), *α* is the significance level (0.05), and 1 − *β* is the power to detect a treatment effect (80%)

*Abbreviations*: *C9orf72* chromosome 9 open reading frame 72, *GRN* progranulin, *MAPT* microtubule-associated protein tau, *ES* effect size as a proportion of the difference between the outcome in the CDR® plus NACC-FTLD 0.5 group and the outcome in the CDR® plus NACC-FTLD 1 group, *D-KEFS CWIT* Delis-Kaplan Executive Function System Colour-Word Interference Test, *FCSRT* Free and Cued Selective Reminding Test

### Time-to-event analysis

Kaplan-Meier curves can be seen in Fig. 1, and details on the sample included in the time-to-event analysis are reported in Additional file 1: Table S4. For *C9orf72* repeat expansion carriers, the probability of converting to a CDR® plus NACC FTLD of ≥ 1 increases from 6% after 2 years (SE = 0.06, 95% CI 0.01–0.39) to 53% after 3 years (SE = 0.33, 95% CI 0.12–0.99). In *GRN* mutation carriers, the probability of converting to a CDR® plus NACC FTLD of ≥ 1 increased from 4% after 1 year (SE = 0.04, 95% CI 0.01–0.24) to 43% after 3 years (SE = 0.14, 95% CI 0.22–0.72). In *MAPT* mutation carriers, the probability of converting to a global score of ≥ 1 increased from 10% after 1 year (SE = 0.10, 95% CI 0.01–0.49) to 42% during the second year (SE = 0.20, 95% CI 0.14–0.85). The Kaplan-Meier curve for *MAPT* mutation increased to 100% after 3 years in Fig. 1 because only one mutation carrier had follow-up up to this point and this individual progressed to a CDR® plus NACC FTLD of ≥ 1. There was no significant difference between the progression rates of different genetic groups (*X*^2^(2) = 1.18, *p* = 0.55). In the total group of mutation carriers, the probability of converting to a CDR® plus NACC FTLD of ≥ 1 was 21% after 2 years (SE = 0.03, 95% CI 0.11–0.40) and 52% after 3 years (SE = 0.16, 95% CI 0.26–0.83). This means that for a 3-year trial where drug treatment is assumed to have s 20% effect (i.e. only 80% of the treated group will experience the change seen between CDR® plus NACC FTLD 0.5 and 1 groups), the sample size corresponding to a 10% effect in Table 3 needs to be included in order to demonstrate a treatment effect, because only ~ 50% of mutation carriers would be expected to progress from CDR® plus NACC FTLD 0.5 to 1 without treatment (i.e. effect size needs to be divided by 2).

## Discussion

We have empirically developed gene-specific cognitive composite scores in *MAPT*, *GRN*, and *C9orf72* mutation carriers (GENFI-Cog) and demonstrated that they provide feasible sample sizes for clinical trials to evaluate the effect of treatment on clinical progression from the prodromal to the fully symptomatic stage. Time-to-event analyses revealed that roughly 50% of the patients with a CDR® plus NACC FTLD of 0.5 progress to 1 or higher after a period of 3 years. The results from this study show that GENFI-Cog has potential as a cognitive endpoint in upcoming clinical trials and provide important guidelines on sample size recruitment and clinical trial duration.

The GENFI-Cog composites can be regarded as attractive clinical outcome measures because they produce substantially lower sample size estimates than most individual neuropsychological tests. Depending on the effect size (40% to 10%), sample size estimates ranged between 13 and 214 for *C9orf72*, 3 and 53 for *GRN*, and 6 and 90 for *MAPT* per study arm for the weighted GENFI-Cog. A practical problem in trial design for FTD spectrum disorders is recruiting enough patients to test candidate therapeutics as FTD is much less common than AD, with an estimated prevalence of 15/100,000 and approximately 10–20% of cases being caused by mutations in *C9orf72*, *GRN*, and *MAPT* genes [4, 7, 42]. It is therefore unlikely that a trial would be able to include many hundreds of patients per study arm, which our results showed would be necessary for most individual neuropsychological tests. There were some individual neuropsychological tests that required reasonable sample sizes similar to that of GENFI-Cog, e.g. TMT and D-KEFS CWIT. These tests are typically included in clinical trials such as the current AL001 study of *GRN*-related FTD [7]. Yet, due to the heterogeneity in cognitive symptoms between patients even with the same genetic mutation, individually examining each cognitive test might not provide a sensitive and clinically meaningful primary outcome measure. Using GENFI-Cog will allow a single cognitive outcome to be used when analysing treatment effect, although validation in other large cohorts is warranted.

The CDR® plus NACC FTLD is currently often used as an inclusion criterion for clinical trials as well as for tracking disease progression. The results showed that roughly 50% of the patients with a CDR® plus NACC FTLD 0.5 progress to 1 or higher after a period of 3 years. This indicates that for trials with a duration of 3 years, around 50% of patients with CDR® plus NACC FTLD of 0.5 on entry to the trial would be expected to progress to CDR® plus NACC FTLD of 1 in the absence of effective disease-modifying treatment. This means that if a treatment is expected to have a 20% effect, the sample size corresponding to a 10% effect needs to be included per study arm to be able to demonstrate a treatment effect, because only half of the mutation carriers would be expected to progress from CDR® plus NACC FTLD 0.5 to 1 without treatment. This is important to consider when planning trial duration and recruitment with the currently available clinical measures.

The optimal gene-specific cognitive composite score incorporated tests from different cognitive domains. For *GRN* mutation carriers, tests for executive function and social cognition contributed the most to the composite score, with the addition of tests for memory and language. In *MAPT* mutation carriers, there was a strong focus on semantic and episodic memory tests in the composite score with the addition of tests for attention and mental processing speed. A combination of tests from all cognitive domains was most sensitive in *C9orf72* mutation carriers, with the strongest contribution from tests within the domains of executive function, social cognition, and memory. These results complement recent studies showing cognitive decline in the early stages of FTD with widespread cognitive impairment covering multiple domains in *C9orf72* [22, 43], dysexecutive functioning as the key feature in *GRN* [13, 22] and a specific impairment in episodic and semantic memory in *MAPT*-associated FTD [13, 20, 22]. Impairment of social cognition appears to be a key feature in all three genetic groups [38], which was probably due to the high number of bvFTD cases in the sample. Neuroimaging studies have indeed shown that the neurodegenerative process in *C9orf72* mutation carriers typically is reflected by widespread degeneration in frontal, temporal, and cerebellar and subcortical structures [43], whereas focal atrophy of the anteromedial temporal lobe, an area important for memory and semantic functioning, is often seen in *MAPT*-associated FTD [44]. In *GRN* mutation carriers, the typical pattern of degeneration includes the inferior frontal regions as well as the cingulate cortex, areas known to be critical in executive function [44]. Thus, although the GENFI-Cog was empirically derived, the selected tests are clinically meaningful and in line with a theoretically driven approach where the composite would be constructed a priori from cognitive tests that are known to decline in the early stages of each genetic group.

This is to our knowledge the first study that has created cognitive composites for genetic forms of FTD by selecting the most sensitive combinations of cognitive variables based on systematic comparisons with controls. A major strength of this study is the use of a large cohort of genetic FTD mutation carriers allowing gene-specific analyses, but also the use of a matched control group of mutation-negative family members. Another strength is the use of LASSO with cross-validation to avoid overfitting bias to ensure that results have generalizability [41].

### Limitations

There are some limitations to the present study, however. The results from the logistic regression analysis revealed two neuropsychological tests in *GRN* mutation carriers with a positive coefficient, indicating better performance compared to controls, and were excluded from the composite scores. Development of GENFI-Cog was constrained by the neuropsychological test battery that is used in the GENFI cohort [14], which made validation in an independent sample not possible and limited the generalizability of the findings. Validation in other cohorts (such as ALLFTD [45] or DINAD) is therefore recommended. Although the LASSO model with 10-fold cross-validation included an internal cross-validation step to select the penalization term for the selection of the cognitive tests, the findings were not externally validated in an independent sample thereby limiting the generalizability of GENFI-Cog. Future collaborations within the FTD Prevention Initiative (FPI) could be a starting point to cross-validate our findings. The sample size estimates serve as a guide on the sensitivity and power of GENFI-Cog compared to individual cognitive tests and should be interpreted with caution as they were calculated from the cross-sectional difference between a small number of patients with CDR® plus NACC FTLD 0.5 and 1, assuming that the difference between these groups is representative of the change over time that would be seen in longitudinal scores in a clinical trial as patients progress from a score of 0.5 to 1, i.e. prodromal to fully symptomatic. Future research using longitudinal data and larger sample sizes is necessary to examine the validity of this assumption and to examine if the cognitive composites presented in the current study are similar to those derived using longitudinal change in scores. Importantly, it is essential for future clinical trials of FTD to also include other biomarkers such as neuroimaging, neurofilament light chain, or other fluid protein levels as endpoints. As such, it would be interesting to include such biomarkers in addition to GENFI-Cog within a future longitudinal multimodal analysis. Lastly, as GENFI is a prospective cohort study with ongoing recruitment, not all participants completed the same number of visits contributing to low sample sizes at later visits in the time-to-event analysis. The time-to-event analysis was performed to provide insight on the possible duration required for a clinical trial, but validation with larger sample sizes where all participants have completed the same number of visits is warranted.

## Conclusions

In summary, we examined the cognitive data from the GENFI cohort and conducted a search for the combination of cognitive assessments most sensitive to differentiate *MAPT*, *GRN*, and *C9orf72* mutation carriers from non-carriers. As a result, we created three gene-specific cognitive composite scores, GENFI-Cog, that were sensitive to track progression on the clinical progression of the CDR® plus NACC FTLD 0.5 to 1 stage as it resulted in smaller sample sizes than most individual neuropsychological tests. To conclude, GENFI-Cog has the potential to be a primary cognitive outcome measure in upcoming clinical trials for *C9orf72*, *GRN*, and *MAPT* mutation carriers.

## Supplementary Information

**Additional file 1: Table S1.** Number of control data available in each language per cognitive test. **Table S2.** Parameters included in the sample size calculations. **Table S3.** Participants characteristics and neuropsychological test results per CDR® plus NACC FTLD global score. **Table S4.** Number of mutation carriers that progressed on the CDR® plus NACC FTLD. **Figure S1.** STROBE flowchart.

## Data Availability

The datasets used and/or analysed during the current study are available from the corresponding author on reasonable request

## Abbreviations

AD: Alzheimer’s disease
ADAS-Cog: Alzheimer’s Disease Assessment Scale Cognitive Subscale
ALLFTD: ARTFL-LEFFTDS Longitudinal Frontotemporal Lobar Degeneration Study
BNT: Boston Naming Test
bvFTD: Behavioural variant frontotemporal dementia
C9orf72: Chromosome 9 open reading frame 72
CDR plus NACC-FTLD: Clinical Dementia Rating scale plus National Alzheimer’s Coordinating Center – Frontotemporal Lobar Degeneration
CWIT: Colour Word Interference Test
DINAD: Dominantly inherited non-Alzheimer dementias
D-KEFS: Delis-Kaplan Execution Function System
FCSRT: Free and Cued Selective Reminding Test
FTD: Frontotemporal dementia
FTD-ALS: Frontotemporal dementia with amyotrophic lateral sclerosis
GENFI: Genetic FTD Initiative
GRN: Progranulin
HD: Huntington’s disease
LASSO: Least absolute shrinkage and selection operator
MAPT: Microtubule-associated protein tau
MMSE: Mini-Mental State Examination
PD: Parkinson’s disease
PPA: Primary progressive aphasia
TMT: Trail Making Test
UPDRS: Unified Parkinson’s Disease Rating Scale
UHDRS: Unified Huntington’s Disease Rating Scale
WAIS-R: Wechsler Adult Intelligence Scale - Revised
WMS-R: Wechsler Memory Scale - Revised
