# Supplementary material for: Cognitive composites for genetic frontotemporal dementia: GENFI-Cog
Source: Alzheimers Res Ther. 2022 Jan 19;14:10. doi: 10.1186/s13195-022-00958-0 (PMC8772227; doi:10.1186/s13195-022-00958-0)
Supplement: Supplementary file 1 — Additional file 1: Table S1. Number of control data available in each language per cognitive test. Table S2. Parameters included in the sample size calculations. Table S3. Participants characteristics and neuropsychological test results per CDR® plus NACC FTLD global score. Table S4. Number of mutation carriers that progressed on the CDR® plus NACC FTLD. Figure S1. STROBE flowchart. [file 13195_2022_958_MOESM1_ESM.docx]

**Poos et al. – Additional file 1**

**Description of statistical analysis**

A.1 Calculating the Z-scores corrected for covariates

Raw data of each cognitive outcome was converted to a Z-score corrected for age, education and sex (and language for the Free and Cued Selective Reminding Test) compared to 312 healthy controls as collected within GENFI (i.e. mutation negative participants). The formula for this calculation is:

Corrected Z-score = [(raw score – predicted score)/standard deviation of the residuals in control group]

A linear regression with all covariates was performed in the control group to calculate the residuals and the coefficients of each covariate. The predicted scores were calculated with the coefficients from the model according to the following formula:

Predicted score = (*a*) + (*b**age) + (*c**education) + (*d**sex) + (*e**language)

A.2 Developing the cognitive composite score

Logistic regression models were used to identify the combination of neuropsychological tests that discriminated best between each mutation carrier group and controls, where mutation carrier status was the outcome and the cognitive tests the predictors of interest. Due to relatively small sample sizes least absolute shrinkage and selection operator (LASSO) regularisation was used in the logistic regression model. This approach adds a penalization term (λ) to the log-likelihood function which forces the sum of the absolute value of the regression coefficients to be less than the fixed value. This has the effect of shrinking all coefficients towards zero (James, Witten, Hastie and Tibshirani, 2013), and can therefore be used to perform variable selection. If λ=0 the results are the same as those from a standard logistic regression model, but as λ is increased, fewer tests are selected in the final model and their coefficients are shrunk towards zero. The optimal size for λ was specified by using 10-fold cross validation to find the value that had the smallest error in prediction of mutation carrier status. This approach divides the data into 10 “folds” of equal size, fits the LASSO logistic regression model using only 9 of the 10 folds, and calculates the prediction error on the remaining “fold” not used in fitting the model. This is repeated excluding each of the 10 folds one at a time and the average prediction error across the 10 folds gives a measure of cross-validated performance. This process was repeated for λ between 0.001 to 100. The final λ was selected as the one which showed minimum cross-validation error in prediction of mutation carrier status (i.e. one which had the most parsimonious model but achieved an error of within 1 standard error of the minimum value). The package glmnet in R was used to fit the LASSO models and perform the cross-validation.

From the resulting model two different cognitive composite scores were calculated: (1) average of the scores for all cognitive tests that were selected in the model; and (2) weighted average of the scores for all cognitive tests that were selected in the model, using the regression coefficients to determine the weights (i.e. individual regression coefficient/sum of coefficients). For example:

Average composite:

$$Score1=\frac{x_{1}+ x_{2}+x_{3}}{3}$$

Weighted composite:

$$core2=\frac{0.25x_{1}+0.15x_{2}+0.10x_{3}}{0.25+0.15+0.10}$$

A.3 Sample size calculation

To evaluate performance of the cognitive composites and all the individual cognitive tests, sample sizes were calculated for a hypothetical clinical trial of a disease modifying treatment. The sample size for a hypothetical two arm study with 1:1 randomization to placebo versus active to have 80% power to detect a treatment effect at 5% significance level was calculated as:

$$n=(1-\rho^{2})\frac{2\sigma^{2}}{\delta^{2}}\left( Z_{\alpha/2}+Z_{\beta} \right)^{2}$$

Where,

ρ is the correlation between baseline and one year follow-up measures of the outcome of interest at baseline in each mutation carrier group. There were 25 *C9orf72*, 19 *GRN* and 13 *MAPT* mutation carriers that had a complete follow-up visit after one year. Pearson correlation analysis was used to estimate the correlation between baseline and follow-up.

σ is the standard deviation of the outcome of interest in the group with CDR® plus NACC FTLD 0.5 at baseline (*C9orf72*: n=28, *GRN*: n=25, *MAPT*: n=13).

δ is the treatment effect as defined below.

α is the type I error rate (significance level) for the two-sided test comparing active treatment to control

β is the type II error rate (100%-power) for the two-sided test comparing active treatment to control

$Z_{\alpha/2}$ is the value of the standard normal distribution above which the proportion of the distribution is equal to α/2

$Z_{\beta}$ is the value of the standard normal distribution above which the proportion of the distribution is equal to β

For 80% power to detect a treatment effect at 5% significance level:

$$\left( Z_{\alpha/2}+Z_{\beta} \right)^{2}=\left( 1.960+0.842 \right)^{2}=7.85$$

Sample sizes were calculated for three hypothesized treatment effects (δ), which were each a proportion (10%, 20% and 40%) of the difference in mean between the CDR® plus NACC FTLD 0.5 and 1 group (*C9orf72*: n=28 and n=14, *GRN*: n=25 and n=10, *MAPT*: n=13 and n=7).

**Table A.1. Number of control data available in each language per cognitive test.**

|  | **English** | **Dutch** | **Spanish** | **French** | **Italian** | **Swedish** | **Portugese** | **German** |
| --- | --- | --- | --- | --- | --- | --- | --- | --- |
| Camel and Cactus Test | 78 | 62 | 38 | 39 | 39 | 15 | 14 | 8 |
| Boston Naming Test | 78 | 79 | 38 | 39 | 38 | 15 | 14 | 7 |
| Category fluency | 78 | 79 | 38 | 39 | 38 | 14 | 14 | 8 |
| Digit span forward | 78 | 80 | 38 | 39 | 40 | 15 | 14 | 8 |
| Trail Making Test - part A | 77 | 79 | 38 | 39 | 39 | 15 | 14 | 8 |
| Digit Symbol | 78 | 65 | 38 | 39 | 38 | 15 | 14 | 8 |
| D-KEFS CWIT - color naming | 78 | 77 | 38 | 39 | 37 | 15 | 14 | 8 |
| D-KEFS CWIT - word naming | 78 | 77 | 38 | 39 | 37 | 15 | 14 | 8 |
| Digit span backward | 78 | 80 | 38 | 39 | 40 | 15 | 14 | 8 |
| Trail Making Test - part B | 77 | 79 | 38 | 37 | 38 | 15 | 14 | 8 |
| D-KEFS CWIT - ink naming | 78 | 77 | 38 | 39 | 37 | 15 | 14 | 8 |
| Phonemic fluency | 77 | 62 | 38 | 39 | 38 | 15 | 13 | 8 |
| Benson Figure copy | 78 | 65 | 38 | 39 | 40 | 15 | 14 | 8 |
| Benson Figure recall | 78 | 65 | 38 | 39 | 40 | 15 | 14 | 8 |
| FCSRT free recall | 77 | 62 | 38 | 39 | 38 | 15 | 13 | 8 |
| FCSRT total recall | 77 | 62 | 38 | 39 | 38 | 15 | 13 | 8 |
| FCSRT delayed free recall | 77 | 62 | 38 | 39 | 38 | 15 | 13 | 8 |
| FCSRT delayed total recall | 77 | 62 | 38 | 39 | 38 | 15 | 13 | 8 |
| Facial Emotion Recognition Test | 78 | 61 | 38 | 39 | 38 | 15 | 14 | 8 |

Abbreviations: D-KEFS CWIT = Delis-Kaplan Executive Function System Color-Word Interference Test; FCSRT = Free and Cued Selective Reminding Test.

**Table A.2 Parameters included in the sample size calculations**

|  | ***C9orf72*** | | | ***GRN*** | | | ***MAPT*** | | |
| --- | --- | --- | --- | --- | --- | --- | --- | --- | --- |
|  | MD | σ | ρ | MD | σ | ρ | MD | σ | ρ |
| **Cognitive composite scores** | | | | | | | | | |
| Average | -1.01 | 0.83 | 0.76 | -1.44 | 0.42 | 0.92 | -1.67 | 1.29 | 0.83 |
| Weighted | -1.13 | 0.74 | 0.77 | -1.70 | 0.66 | 0.85 | -2.12 | 1.87 | 0.86 |
| **Language** | | | | | | | | | |
| Camel and Cactus Test | -0.71 | 2.87 | 0.45 | -0.94 | 0.69 | 0.76 | -1.47 | 2.28 | 0.72 |
| Boston Naming Test | -1.36 | 3.49 | 0.63 | -1.49 | 0.57 | 0.47 | -1.91 | 2.88 | 0.82 |
| Category fluency | -0.75 | 0.80 | 0.29 | -0.97 | 0.70 | 0.33 | -0.66 | 0.36 | 0.69 |
| **Attention and mental processing speed** | | | | | | | | | |
| Digit span forward | -0.07 | 0.93 | 0.57 | -0.68 | 1.34 | 0.41 | 0.22 | 1.36 | 0.61 |
| Trail Making Test - part A | -0.58 | 1.88 | 0.74 | -1.80 | 0.58 | 0.84 | -1.05 | 0.38 | 0.87 |
| Digit Symbol | -1.17 | 1.11 | 0.80 | -1.13 | 1.14 | 0.34 | -1.26 | 1.08 | 0.93 |
| D-KEFS CWIT - color naming | -1.24 | 3.06 | 0.72 | -1.49 | 1.06 | 0.32 | -1.99 | 0.79 | 0.79 |
| D-KEFS CWIT - word naming | -0.26 | 1.83 | 0.56 | -0.52 | 0.83 | 0.32 | -0.87 | 0.41 | 0.82 |
| **Executive function** | | | | | | | | | |
| Digit span backward | -0.85 | 1.46 | 0.46 | -1.06 | 1.03 | 0.42 | -0.17 | 0.77 | 0.38 |
| Trail Making Test - part B | -1.43 | 4.14 | 0.60 | -3.51 | 2.30 | 0.92 | -2.07 | 0.75 | 0.70 |
| D-KEFS CWIT - ink naming | -2.31 | 1.52 | 0.86 | -2.84 | 0.87 | 0.59 | -1.94 | 0.47 | 0.86 |
| Phonemic fluency | -0.80 | 1.25 | 0.82 | -0.54 | 0.85 | 0.51 | -1.22 | 0.88 | 0.83 |
| **Visuoconstruction** | | | | | | | | | |
| Benson Figure copy | -0.71 | 2.71 | 0.30 | -0.64 | 0.57 | 0.03 | -0.02 | 0.95 | 0.00 |
| **Memory** | | | | | | | | | |
| Benson Figure recall | -0.74 | 0.87 | 0.58 | 1.38 | 1.22 | 0.35 | -0.77 | 2.93 | 0.01 |
| FCSRT free recall | -0.93 | 1.15 | 0.38 | -0.87 | 0.92 | 0.85 | -1.68 | 2.20 | 0.58 |
| FCSRT total recall | -1.80 | 4.57 | 0.54 | -0.72 | 0.96 | 0.83 | -3.11 | 5.98 | 0.46 |
| FCSRT delayed free recall | -1.10 | 1.19 | 0.61 | -0.81 | 1.42 | 0.77 | -1.86 | 2.39 | 0.76 |
| FCSRT delayed total recall | -2.07 | 2.99 | 0.67 | -0.60 | 1.28 | 0.97 | -3.02 | 8.38 | 0.53 |
| **Social** **cognition** | | | | | | | | | |
| Facial Emotion Recognition Test | -0.47 | 1.66 | 0.37 | -0.41 | 1.55 | 0.47 | -1.68 | 1.42 | 0.81 |

MD is the mean difference between the CDR® plus NACC-FTLD 0.5 and 1 group; σ is the standard deviation of the outcome in the CDR® plus NACC-FTLD 0.5 group; ρ is the correlation between baseline and follow-up measures of the outcome. Abbreviations: *C9orf72* = chromosome 9 open reading frame 72; *GRN* = progranulin; *MAPT* = microtubule-associated protein tau; CDR® plus NACC FTLD = Clinical Dementia Rating scale plus National Alzheimer’s Coordinating Center Frontotemporal Lobar Degeneration; D-KEFS CWIT = Delis-Kaplan Executive Function System Color-Word Interference Test; FCSRT = Free and Cued Selective Reminding Test.

**Table A.3. Participants characteristics and neuropsychological test results per CDR® plus NACC FTLD global score.**

|  | ***C9orf72*** | | | | ***GRN*** | | | | ***MAPT*** | | | |
| --- | --- | --- | --- | --- | --- | --- | --- | --- | --- | --- | --- | --- |
| CDR® plus NACC FTLD global score | 0.5 | 1 | 2 | 3 | 0.5 | 1 | 2 | 3 | 0.5 | 1 | 2 | 3 |
| Number of participants | 28 | 14 | 18 | 9 | 25 | 10 | 3 | 3 | 13 | 7 | 5 | 3 |
| Sex f:m | 17:11 | 4:10 | 5:13 | 4:5 | 13:12 | 6:4 | 1:2 | 0:3 | 9:4 | 3:4 | 0:5 | 2:1 |
| Age | 47.26 (11.31) | 56.99 (11.32) | 62.33 (8.91) | 61.31 (5.87) | 48.24 (11.39) | 58.63 (6.62) | 66.36 (4.97) | 61.02 (0.63) | 44.28 (11.84) | 52.62 (12.72) | 59.62 (7.46) | 62.81 (3.10) |
| Education | 14.43 (2.64) | 13.57 (3.57) | 13.72 (2.93) | 11.56 (3.13) | 14.92 (3.41) | 13.00 (4.00) | 12.33 (3.06) | 12.00 (1.73) | 13.77 (2.31) | 14.71 (3.50) | 12.40 (4.51) | 18.67 (2.31) |
| MMSE | 28.79 (2.01) | 27.57 (1.87) | 25.56 (2.57) | 24.25 (5.57) | 28.08 (6.16) | 24.50 (9.37) | 26.0 (3.0) | 19.50 (0.71) | 28.50 (2.15) | 27.83 (2.56) | 25.60 (4.51) | 25.67 (2.89) |
| CDR® plus NACC FTLD sum of boxes | 1.07 (0.75) | 4.14 (1.60) | 9.78 (2.61) | 16.06 (2.08) | 0.82 (0.64) | 3.50 (0.91) | 11.17 (3.01) | 16.83 (1.26) | 1.08 (0.76) | 3.57 (1.37) | 9.80 (0.76) | 15.17 (2.57) |
| **Language** | | | | | | | | | | | | |
| Camel and Cactus Test | -0.58 (1.70) | -1.30 (2.25) | -3.14 (2.44) | -3.79 (4.73) | -0.16 (0.83) | -1.11 (1.82) | -1.10 (2.58) | -1.69 (1.08) | -0.45 (1.51) | -1.92 (2.91) | -4.77 (2.83) | -5.26 (4.70) |
| Boston Naming Test | -0.23 (1.87) | -1.58 (3.49) | -2.88 (2.29) | -4.64 (5.54) | -0.05 (0.75) | -1.44 (1.61) | -1.24 (1.44) | -2.84 (4.10) | -0.76 (1.70) | -2.66 (2.45) | -4.16 (2.83) | -8.11 (3.10) |
| Category fluency | -0.46 (0.90) | -1.21 (0.81) | -1.96 (0.75) | -1.97 (0.74) | -0.04 (0.84) | -1.01 (0.64) | -1.21 (0.74) | -2.46 (0.61) | -0.23 (0.60) | -0.89 (1.33) | -1.57 (0.91) | -2.14 (1.47) |
| **Attention and mental processing speed** | | | | | | | | | | | | |
| Digit span forward | 0.06 (0.96) | -0.01 (1.10) | -1.01 (1.20) | -1.16 (1.16) | 0.16 (1.16) | -0.52 (1.46) | 0.06 (0.70) | -0.80 (1.68) | -0.02 (1.17) | 0.20 (1.02) | 0.04 (1.64) | 0.75 (1.73) |
| Trail Making Test - part A | -0.45 (1.37) | -1.03 (2.12) | -2.31 (1.96) | -2.87 (3.25) | 0.03 (0.76) | -1.83 (1.99) | -3.48 (1.40) | -0.11 (1.01) | 0.25 (0.62) | -1.30 (1.35) | -1.06 (1.36) | -3.02 (2.28) |
| Digit Symbol | -0.23 (1.05) | -1.40 (1.02) | -2.11 (0.89) | -1.97 (1.16) | -0.17 (1.07) | -1.30 (1.41) | -1.50 (0.60) | -1.19 (0.81) | 0.02 (1.04) | -1.23 (1.61) | -0.76 (0.40) | -2.21 (0.94) |
| D-KEFS CWIT - color naming | -0.70 (1.75) | -1.94 (1.95) | -5.46 (4.01) | -5.74 (3.96) | -0.04 (1.03) | -1.53 (3.13) | -0.18 (1.05) | -1.53 (0.99) | -0.14 (0.89) | -2.13 (2.65) | -2.95 (2.54) | -1.62 (2.57) |
| D-KEFS CWIT - word naming | -0.42 (1.35) | -0.67 (1.59) | -3.60 (3.42) | -4.73 (4.59) | 0.36 (0.91) | -0.87 (2.40) | 0.43 (0.45) | -0.22 (1.01) | 0.27 (0.64) | -1.15 (1.75) | -1.14 (1.31) | -1.63 (2.41) |
| **Executive function** | | | | | | | | | | | | |
| Digit span backward | 0.19 (1.21) | -0.67 (1.18) | -1.12 (0.85) | -1.40 (0.85) | -0.01 (1.01) | -1.07 (1.44) | -1.31 (0.09) | -1.77 (0.92) | -0.22 (0.88) | -0.39 (0.97) | 0.34 (1.41) | -0.46 (0.80) |
| Trail Making Test - part B | -0.70 (2.04) | -2.13 (2.70) | -4.86 (2.58) | -3.47 (2.94) | -0.27 (1.52) | -3.78 (3.50) | -5.05 (3.35) | -4.85 (3.82) | 0.11 (0.87) | -2.18 (3.01) | -2.34 (2.71) | -4.25 (3.48) |
| D-KEFS CWIT- ink naming | -0.75 (1.23) | -3.06 (3.77) | -6.46 (3.38) | -6.49 (4.60) | -0.28 (0.94) | -3.12 (3.56) | -1.81 (0.61) | -0.86 (0.47) | 0.27 (0.68) | -2.21 (3.40) | -2.36 (2.73) | -2.88 (3.05) |
| Phonemic fluency | -0.45 (1.12) | -1.25 (0.97) | -2.00 (0.79) | -1.70 (1.07) | 0.54 (0.92) | -0.97 (1.54) | -0.80 (0.48) | -1.58 (1.16) | 0.08 (0.94) | -1.14 (1.19) | -0.85 (1.29) | -2.26 (0.97) |
| **Visuoconstruction** | | | | | | | | | | | | |
| Benson Figure copy | -0.29 (1.65) | -0.99 (1.43) | -1.38 (2.14) | -1.68 (2.45) | 0.30 (0.75) | -0.34 (1.17) | -0.49 (0.27) | -1.73 (2.77) | -0.22 (0.98) | -0.24 (1.19) | -0.68 (1.86) | -1.61 (2.60) |
| **Memory** | | | | | | | | | | | | |
| Benson Figure recall | 0.18 (0.93) | -0.57 (1.36) | -1.67 (1.50) | -1.85 (1.98) | -0.28 (1.11) | -1.09 (1.14) | -1.70 (2.21) | -2.51 (2.83) | -0.69 (1.71) | -1.46 (2.12) | -1.34 (2.06) | -3.18 (1.34) |
| FCSRT free recall | -0.87 (1.07) | -1.80 (1.14) | -2.29 (1.21) | -2.77 (1.44) | -0.13 (0.96) | -1.00 (1.30) | -2.43 (1.82) | -2.96 (2.40) | -0.60 (1.48) | -2.28 (1.65) | -2.60 (1.20) | -3.70 (1.53) |
| FCSRT total recall | -0.73 (2.14) | -2.53 (3.37) | -3.31 (3.97) | -4.03 (5.12) | -0.32 (0.98) | -1.05 (1.85) | -5.30 (5.12) | -7.97 (5.55) | -0.92 (2.45) | -4.03 (2.88) | -4.29 (2.80) | -6.17 (7.09) |
| FCSRT delayed free recall | -0.52 (1.09) | -1.63 (1.43) | -2.47 (1.37) | -3.08 (1.34) | -0.43 (1.19) | -1.24 (1.54) | -2.65 (1.59) | -2.89 (2.45) | -0.45 (1.55) | -2.31 (1.85) | -2.65 (1.64) | -4.32 (1.53) |
| FCSRT delayed total recall | -0.34 (1.73) | -2.41 (3.69) | -3.36 (4.30) | -4.59 (5.49) | -0.19 (1.3) | -0.79 (1.84) | -3.40 (5.95) | -7.79 (6.27) | -0.98 (2.89) | -4.00 (4.13) | -4.16 (3.12) | -5.88 (7.20) |
| **Social cognition** | | | | | | | | | | | | |
| Facial Emotion Recognition Test | -0.81 (1.29) | -1.27 (1.11) | -2.49 (1.45) | -3.35 (3.19) | -0.55 (1.24) | -0.96 (1.06) | -2.53 (0.89) | -3.41 (1.99) | -0.56 (1.19) | -1.10 (1.85) | -1.69 (2.27) | -1.88 (1.07) |

Values are: mean Z-scores (raw score – mean score controls/standard deviation of controls) corrected for age, years of education and sex, with standard deviation between parentheses unless otherwise specified. For the FCSRT and letter fluency an additional correction was made for language as stimuli differed between languages. Abbreviations: *C9orf72* = chromosome 9 open reading frame 72; *GRN* = progranulin; *MAPT* = microtubule-associated protein tau; CDR® plus NACC FTLD = Clinical Dementia Rating scale plus National Alzheimer’s Coordinating Center Frontotemporal Lobar Degeneration; D-KEFS CWIT = Delis-Kaplan Executive Function System Color-Word Interference Test; FCSRT = Free and Cued Selective Reminding Test.

**Table A.4.** Number of mutation carriers that progressed on the CDR® plus NACC FTLD.

|  | | Number that did not progress to  CDR® plus NACC FTLD > 0.5 | Number that progressed to CDR® plus NACC FTLD > 0.5 | Number that did not complete visit* |
| --- | --- | --- | --- | --- |
| *C9orf72* | Year 0 | 21 | 0 | 0 |
|  | Year 1 | 16 | 1 | 4 |
|  | Year 2 | 3 | 0 | 11 |
|  | Year 3 | 2 | 1 | 2 |
| *GRN* | Year 0 | 27 | 0 | 0 |
|  | Year 1 | 23 | 1 | 3 |
|  | Year 2 | 7 | 5 | 11 |
|  | Year 3 | 1 | 1 | 6 |
| *MAPT* | Year 0 | 16 | 0 | 0 |
|  | Year 1 | 14 | 0 | 2 |
|  | Year 2 | 7 | 1 | 6 |
|  | Year 3 | 0 | 3 | 4 |

Abbreviations: *C9orf72* = chromosome 9 open reading frame 72; *GRN* = progranulin; *MAPT* = microtubule-associated protein tau; CDR® plus NACC FTLD = Clinical Dementia Rating scale plus National Alzheimer’s Coordinating Center Frontotemporal Lobar Degeneration. *Refers to participants that dropped out of GENFI and participants that had not completed the visit for this study yet. As the data concerns an ongoing prospective cohort study not all participants completed a second, third or fourth visit yet.

Figure A.1. STROBE flowchart


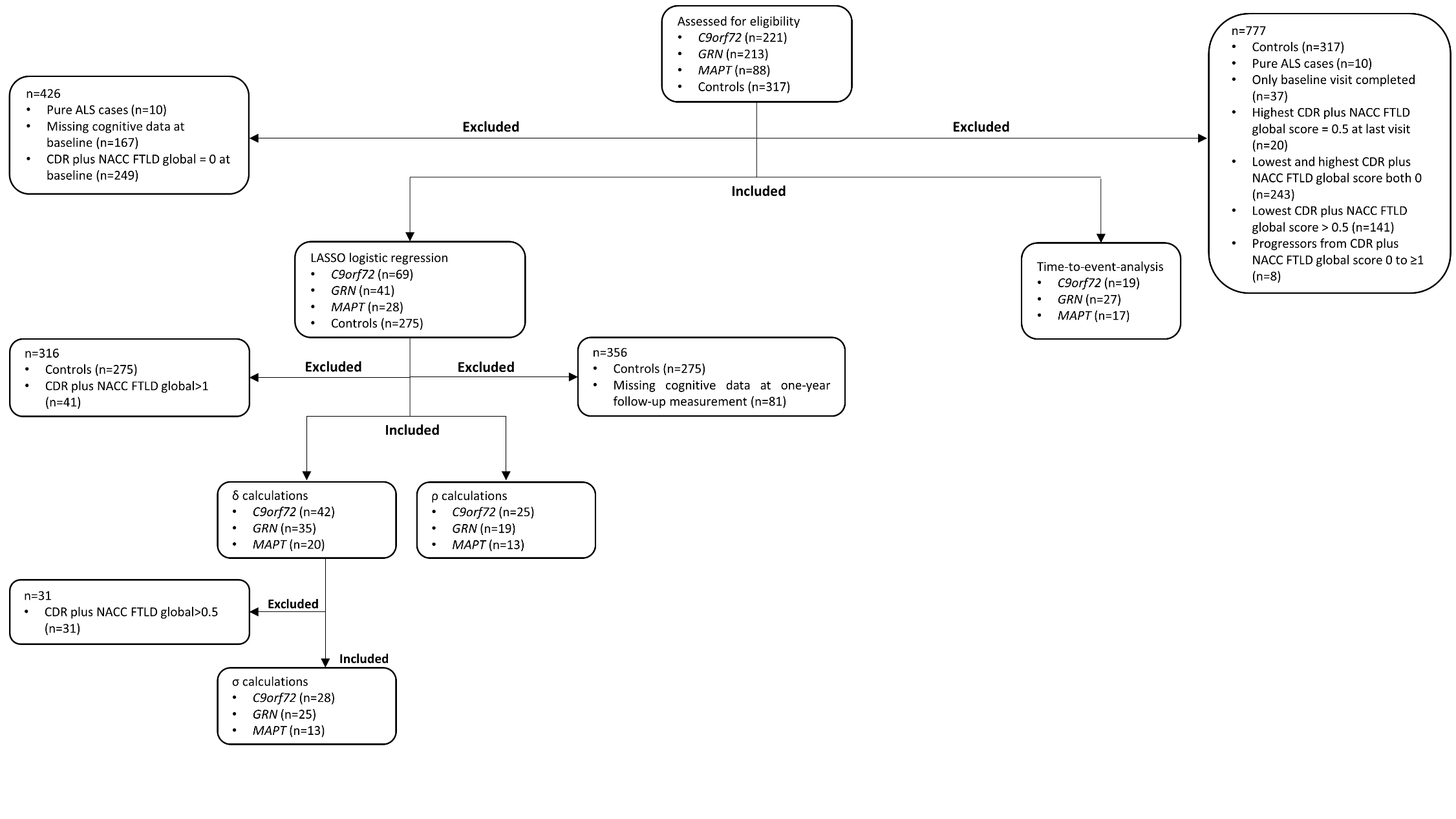


Abbreviations: *C9orf72* = chromosome 9 open reading frame 72; *GRN* = progranulin; *MAPT* = microtubule-associated protein tau; LASSO = least absolute shrinkage and selection operator; CDR plus NACC FTLD = Clinical Dementia Rating scale plus National Alzheimer’s Coordinating Center Frontotemporal Lobar Degeneration. ρ, σ and δ are used for the sample size calculations where, ρ is the correlation between baseline and follow-up measures of the outcome, σ is the standard deviation of the outcome in the CDR® plus NACC-FTLD 0.5 group, δ is the treatment effect (effect size multiplied by difference in mean between CDR® plus NACC-FTLD 0.5 and 1 group).
